# Low-density lipoprotein particle profiles compared with standard lipids measurements in the association with asymptomatic intracranial artery stenosis

**DOI:** 10.1038/s41598-024-59523-4

**Published:** 2024-05-10

**Authors:** Thien Vu, Yuichiro Yano, Huy Kien Tai Pham, Rajib Mondal, Mizuki Ohashi, Kaori Kitaoka, Mohammad Moniruzzaman, Sayuki Torii, Akihiko Shiino, Atsushi Tsuji, Takashi Hisamatsu, Tomonori Okamura, Keiko Kondo, Aya Kadota, Yoshiyuki Watanabe, Kazuhiko Nozaki, Hirotsugu Ueshima, Katsuyuki Miura

**Affiliations:** 1 NCD Epidemiology Research Center, Shiga University of Medical Science, Otsu, Shiga Japan; 2 Department of Cardiac Surgery, Cardiovascular Center, Cho Ray Hospital, Ho Chi Minh City, Vietnam; 3 Department of Oriental Internal Medicine, Faculty of Traditional Medicine, University of Medicine and Pharmacy, Ho Chi Minh City, Vietnam; 4 Department of Geriatrics, Gia-Dinh People’s Hospital, Ho Chi Minh City, Vietnam; 5 Department of Public Health, Shiga University of Medical Science, Otsu, Shiga Japan; 6 Molecular Neuroscience Research Center, Shiga University of Medical Science, Otsu, Shiga Japan; 7 Department of Neurosurgery, Shiga University of Medical Science, Otsu, Shiga Japan; 8 Department of Public Health, Okayama University Graduate School of Medicine, Dentistry and Pharmaceutical Sciences, Okayama, Japan; 9 Department of Hygiene and Public Health, Keio University School of Medicine, Tokyo, Japan; 10 Department of Radiology, Shiga University of Medical Science, Otsu, Shiga Japan; 11 Higashi-Ohmi General Medical Center, Higashiomi, Shiga Japan

**Keywords:** Low-density lipoprotein particle (LDL-p), Low-density lipoprotein cholesterol (LDL-c), Intracranial artery stenosis (ICAS), Biomarkers, Diseases, Medical research, Risk factors

## Abstract

The Shiga Epidemiological Study of Subclinical Atherosclerosis was conducted in Kusatsu City, Shiga, Japan, from 2006 to 2008. Participants were measured for LDL-p through nuclear magnetic resonance technology. 740 men participated in follow-up and underwent 1.5 T brain magnetic resonance angiography from 2012 to 2015. Participants were categorized as no-ICAS, and ICAS consisted of mild-ICAS (1 to < 50%) and severe-ICAS (≥ 50%) in any of the arteries examined. After exclusion criteria, 711 men left for analysis, we used multiple logistic regression to examine the association between lipid profiles and ICAS prevalence. Among the study participants, 205 individuals (28.8%) had ICAS, while 144 individuals (20.3%) demonstrated discordance between LDL-c and LDL-p levels. The discordance “low LDL-c–high LDL-p” group had the highest ICAS risk with an adjusted OR (95% CI) of 2.78 (1.55–5.00) in the reference of the concordance “low LDL-c–low LDL-p” group. This was followed by the concordance “high LDL-c–high LDL-p” group of 2.56 (1.69–3.85) and the discordance “high LDL-c–low LDL-p” group of 2.40 (1.29–4.46). These findings suggest that evaluating LDL-p levels alongside LDL-c may aid in identifying adults at a higher risk for ICAS.

## Introduction

Intracranial artery stenosis (ICAS), which occurs when plaque builds up in blood vessels at the base of the brain, is linked to stroke and cognitive decline worldwide^1^. ICAS causes about 5–10% of strokes in the White population and up to 30–50% of strokes in Asian people^2^. Recognizing and controlling the risk factors for asymptomatic ICAS at an early stage is crucial for delaying the evolution of the disease, which may lead to preventing stroke and cognitive decline.

Low-density lipoprotein cholesterol (LDL-c) is a widely-accepted marker of LDL, and well-recognized as one of the most important causal factors in both the onset and progression of cardiovascular disease^3^. However, approximately 14% of patients with severe ICAS experienced recurrent ischemic stroke within a year, despite exhibiting reduced LDL-c levels due to statin treatment^4^. The use of additional metrics for assessing LDL, such as LDL particles (LDL-p), may potentially provide a more comprehensive understanding of this residual risk.

Nuclear magnetic resonance (NMR) spectroscopy is a technique that enables the simultaneous quantification of both the size and concentration of lipoprotein particles. It has been observed that the NMR-measured LDL-p is closely associated with the initiation and progression of cardiovascular diseases, and its association is either comparable or even stronger than that of the corresponding LDL-c^5–8^. Nevertheless, there is a lack of studies examining the association between NMR-measured LDL-p and ICAS in the Asian population. Moreover, the precise clinical implications of the discrepancies observed between LDL-c levels and LDL-p with respect to intracranial arteries remains incomplete.

We conducted this cross-sectional study in a well-defined community-based Japanese cohort to determine the association of conventional LDL-c and NMR-measured LDL-p with the prevalence of ICAS. We also evaluated the proportion of individuals within the population who exhibited a discordance between LDL-c and LDL-p levels, and their corresponding risk for developing ICAS among healthy Japanese men.

## Methods

### Study population

The Shiga Epidemiological Study of Subclinical Atherosclerosis (SESSA) is a prospective population-based study conducted in Japan. Previous publications have provided a thorough explanation of the study's design and the procedures for recruiting participants^9,10^. Briefly stated, from 2006 to 2008, we invited 2379 Japanese men aged 40 to 79 who were inhabitants of Kusatsu city, Shiga, Japan, at random, based on the Basic Residents’ Register of the city. A total of 1094 males (a participation rate of 46%) participated in the study. Between 2012 and 2015, participants in the baseline survey were invited to engage in follow-up survey, which included an MRI scan. 740 males participated in both the baseline survey and the survey of the MRI scan. Written informed consent was obtained from all individuals.

We included the 711 participants who were remained for analysis after excluding the 28 participants who had any documented history of myocardial infarction or stroke and the 1 participant who had pertinent data missing. The study complies with the ethical principles defined in the Declaration of Helsinki and obtained approval from the Institutional Review Board at Shiga University of Medical Science in Otsu, Japan (No. R2008-061). All participants provided written informed consent. The study followed the STROBE standards for reporting observational studies, as outlined in the Supplementary^11^.

### Measurements

Each participant completed a self-administered questionnaire to provide information about their medical history and lifestyle characteristics. Trained technicians subsequently verified the accuracy and completeness of the questionnaire with the participants.

Data on several metrics such as height, weight, blood pressure, and other relevant parameters have been collected. The blood pressure was assessed using an automated sphygmomanometer (BP-8800; Omron Colin, Tokyo, Japan).

The calculation of body mass index (BMI) involved dividing weight (kg) by the square of height (m^2^). Hypertension was defined as systolic blood pressure (SBP) ≥ 140 mmHg, diastolic blood pressure (DBP) ≥ 90 mmHg, or taking antihypertensive medications.

Blood samples were obtained during the initial phase of the clinic visit following a 12-h period of fasting^9,10^. The serum was separated using centrifugation at a speed of 3000 revolutions per minute for a duration of 15 min at a temperature of 4 °C, all completed within a time frame of 90 min. A part of the samples was utilized for the purpose of conducting routine laboratory analyses, including tests for standard lipids and glucose. We estimated LDL-c levels with TG < 400 mg/dL using Friedewald's formula^12^. LDL-c values were not calculated for individuals with extremely high TG levels due to the unreliable nature of the Friedewald equation in this context. Instead, we will specify that LDL-c values were reported as missing for these participants.

The rest of the serum samples were kept at − 80 °C. Subsequently, a subset of the specimens was sent under dry ice conditions to LipoScience Inc. (now LabCorp) located in Raleigh, NC, USA, for the purpose of conducting LDL particle measurements using NMR technology. Nuclear magnetic resonance (NMR) spectroscopy was employed in order to quantitatively determine the particle concentrations of low-density lipoprotein (LDL). Additionally, particle concentrations were further determined for 3 LDL subclasses (intermediate-density lipoprotein [IDL], 23–27 nm; large, 21.3–23 nm; small, 18.3–21.2 nm) [5–8]^13,14^. According to the Japanese Diabetes Society (JDS) standard, latex agglutination assays detected HbA1c. We then transformed JDS values to National Glycohemoglobin Standardization Program (NGSP) values using the JDS-recommended formula: HbA1c (NGSP) = 1.02 × HbA1c (JDS) + 0.25 (%)^15^.

Diabetes mellitus was identified with fasting plasma glucose ≥ 126 mg/dL, HbA1c ≥ 6.5%, or taking diabetic medication. Dyslipidemia was established when TG ≥ 150 mg/dL, LDL ≥ 140 mg/dL, HDL < 40 mg/dL, or taking lipid-lowering medication.

It is important to note that all measurements utilized in this study were mentioned above, including demographics, lipid profiles, and covariates, were collected during the baseline phase conducted from 2006 to 2008.

### Intracranial artery stenosis

The brain MRI and Magnetic Resonance Angiography (MRA) were conducted between 2012 and 2015, using a 1.5-Tesla MRI scanner (Signa HDxt 1.5 T ver. 16; GE Healthcare, Milwaukee, Wisconsin). In order to diagnose cerebral small vessel disease, a series of imaging techniques were employed. These included the acquisition of three-dimensional T1-weighted spoiled gradient-recalled (SPGR), two-dimensional T2- and T2*-weighted, fluid-attenuated inversion-recovery (FLAIR), and time-of-flight (TOF) MRA images. The acquisition of T2- and T2*-weighted as well as FLAIR images was performed using a slice thickness of 4 mm without any inter-slice gaps. Two neurosurgeons, who are certified by the Japan Neurosurgery Society, conducted a thorough evaluation of all pictures acquired using MRA/MRI. This assessment was done in triplicate, and the neurosurgeons were unaware of the features of the participants. Discrepancies in evaluations were resolved through the process of adjudication facilitated by neurosurgeons.

The study evaluated the presence of intracranial artery stenosis (ICAS) in a total of 11 intracranial arteries. These arteries included the basilar artery and five bilateral vessels, specifically the intracranial segments of the internal carotid artery (ICA), middle cerebral artery (MCA), anterior cerebral artery (ACA), intracranial segments of the vertebral artery, and the posterior cerebral artery (PCA). According to the criteria used in the Warfarin-Aspirin Symptomatic Intracranial Disease trial, there are four categories of arterial stenosis: no visible stenosis, stenosis ranging from 1 to 49%, stenosis ranging from 50 to 99%, and full blockage at 100%^16^. The categorization of ICAS was established as a dichotomous variable, differentiating between the absence of observable stenosis and the presence of stenosis.

### Statistical analysis

The characteristics of the participants are presented by means and standard deviations (SDs) or medians and interquartile ranges (Q1–Q3) for continuous variables, and percentages for categorical variables. Discrepancies in characteristics by the presence or absence of ICAS and concordance–discordance groups were evaluated using the Student’s t-test, Wilcoxon rank sums test, ANOVA test, Kruskal–Wallis test, or Chi-squared test, as appropriate.

In the primary analysis, binary logistic regression analysis was used to assess the association between lipid indices and ICAS. The models were adjusted for several baseline covariates, including age (years), BMI (kg/m^2^), smoking status (current, past, never), drinking status (current, past, never), hypertension (yes/no), diabetes mellitus (yes/no), and dyslipidemia (yes/no). For comparison purposes, LDL-p model was further adjusted for LDL-c, and vice versa. The adjusted odds ratios (ORs) and 95% confidence intervals (CIs) for the presence of ICAS were computed per 1SD higher of lipid indices.

In sensitivity analyses, we divided each lipid measure into quartiles, and computed multivariable adjusted odds ratios (ORs) and 95% confidence intervals (CIs) of the prevalent AVC, using logistic regression, for the upper 3 quartiles (Q2–Q4) in reference to the lowest quartile (Q1). These logistic regression models were full-adjusted with the mentioned-above covariates.

**Figure 1:**
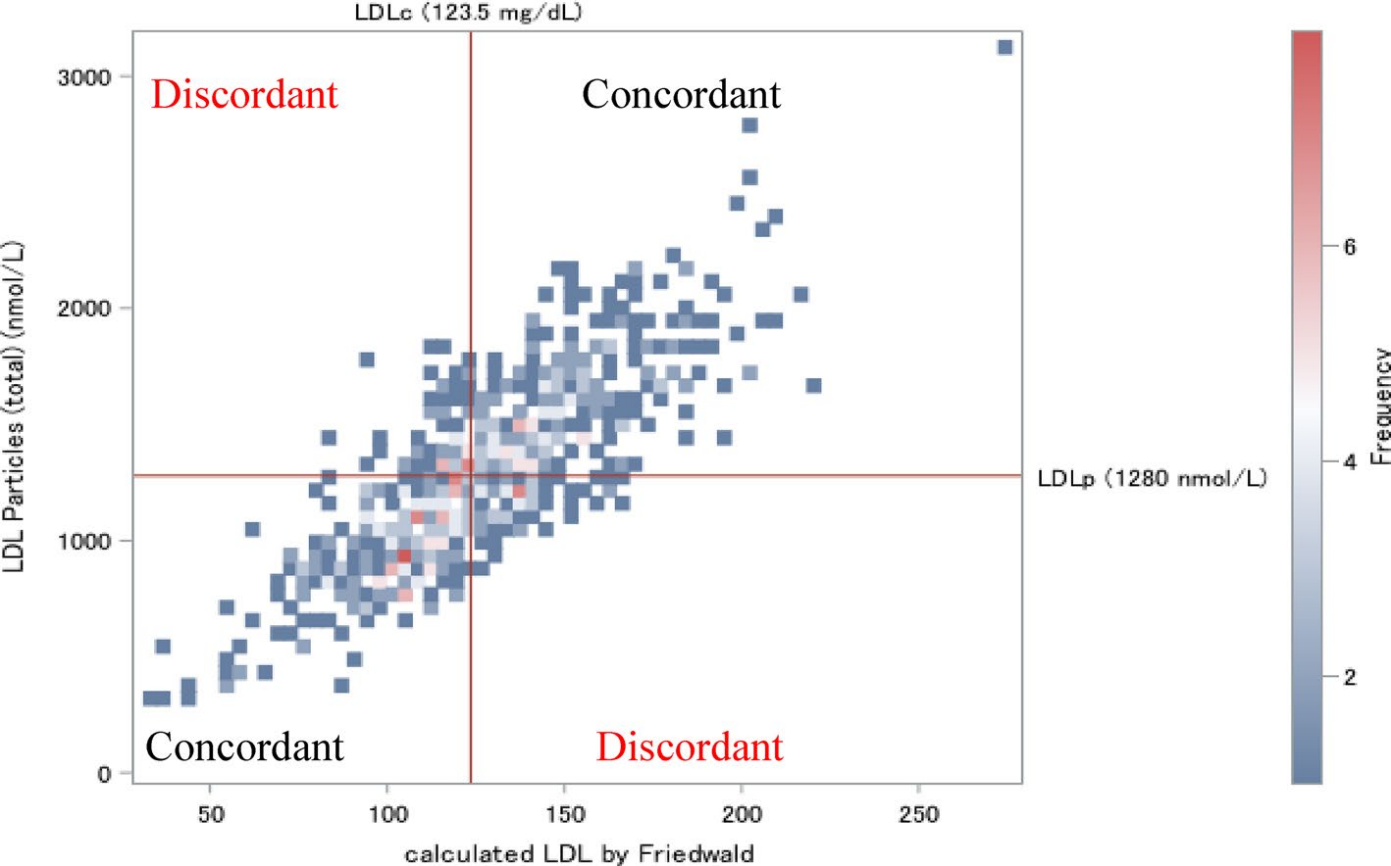
The groups of concordance–discordance between LDL-c and LDL-p based on median value.

To investigate the extent to which discordance or concordance between LDL-c and NMR-measured LDL-p was associated with risk, we classified participants according to median levels of LDL-c (123.5 mg/dL) and LDL-p (1280 nmol/L). LDL-c greater than or equal to the median and the alternative measure LDL-p less than the median, or vice versa, was defined as discordance. Our participants are divided into four groups: concordance “low LDL-c–low LDL-p”, concordance “high LDL-c–high LDL-p”, discordance “low LDL-c–high LDL-p”, and discordance “high LDL-c–low LDL-p” (Fig. 1). We opted for median cut-off points to define concordance–discordance, as there is no physiological cut-off point for this classification. This approach aligns with recommendations by Mora S et al. to facilitate its clinical applicability^17^. We calculated the ORs and 95% CIs for the prevalence of ICAS in the concordance–discordance groups in the reference of concordance “low LDL-c–low LDL-p” using logistic regression with full adjusted for above-mentioned base covariates.

Analyses were performed using SAS version 9.4 (SAS Institute, Cary, NC, USA). Two-tailed p-value < 0.05 was considered significant.

## Results

The characteristics of participants with and without ICAS were presented in Table 1. Among the participants, 205 individuals (28.8%) had ICAS, whereas 506 individuals (71.2%) did not. The participants with ICAS were distinguished by older age, elevated LDL-c, LDL-p, and small LDL-p concentrations, as well as a higher prevalence of hypertension, diabetes, and dyslipidemia compared to those without ICAS. In contrast, the ICAS group had lower HDL-c levels.

**Table 1:** Characteristics of study participants with and without intracranial artery stenosis (ICAS) (Japanese men, aged 40–79, SESSA).

Characteristics	Overall	ICAS (n, %)		*p*-value
	N = 711	Without (506, 71.2)	With (205, 28.8)	
Age, years	64.1 (58.4–71.1)	63.5 (57.3–69.3)	67 (60.2–73.6)	< 0.001
Smoking status, n (%)				0.740
Current	209 (29.4)	152 (30.0)	57 (27.8)	
Past	362 (50.9)	253 (50.0)	109 (53.2)	
Never	140 (19.7)	101 (20.0)	39 (19.0)	
Drinking status, n (%)				0.032
Current	563 (79.2)	413 (81.6)	150 (73.2)	
Past	37 (5.2)	25 (4.9)	12 (5.9)	
Never	111 (15.6)	68 (13.5)	43 (20.9)	
BMI, kg/m^2^	23.6 ± 2.8	23.5 ± 2.8	23.9 ± 2.9	0.091
SBP, mmHg	135.5 ± 18.0	132.9 ± 17.5	142.2 ± 17.7	< 0.001
DBP, mmHg	80.1 ± 10.8	79.7 ± 10.7	80.9 ± 11.1	0.173
Hypertension, n (%)	374 (52.6)	235 (46.4)	139 (67.8)	< 0.001
HbA1c, %	5.4 (5.2–5.9)	5.4 (5.2–5.8)	5.5 (5.3–6.0)	0.001
Glucose, mg/dL	97 (91–107)	97.5 (91–106)	97 (91–111)	0.419
Diabetes mellitus, n (%)	155 (21.8)	99 (19.6)	56 (27.3)	0.023
Dyslipidemia, n (%)	397 (55.8)	261 (51.6)	136 (66.3)	< 0.001
Total cholesterol, mg/dL	210.6 ± 32.5	209.3 ± 32.4	214.1 ± 32.7	0.076
Triglycerides, mg/dL	104 (76–150)	101 (76–47)	114 (78–57)	0.104
HDL-c, mg/dL	59.3 ± 17.1	61.3 ± 17.7	54.6 ± 14.2	< 0.001
LDL-c, mg/dL	126.6 ± 30.8	123.7 ± 30.6	133.9 ± 30	< 0.001
LDL-p, nmol/L	1299.7 ± 392.1	1255.4 ± 386.3	1408.8 ± 385.7	< 0.001
IDL-p, nmol/L	99 (44–170)	99 (42–169)	97 (52–180)	0.477
Large LDL-p, nmol/L	628.3 ± 298.5	621.8 ± 294.9	644.3 ± 307.3	0.364
Small LDL-p, nmol/L	549.3 ± 419.2	513.1 ± 405.3	638.5 ± 440.1	< 0.001

ICAS was diagnosed by differentiation between the absence of detectable stenosis and the presence of stenosis.

Values are presented as mean ± SD for continuous variables with approximately normally distribution or by median (Q1–Q3) with skewed distribution and n (%) for categorical variables. Differences in characteristics were evaluated by using the Student’s t-test, Wilcoxon rank sums test, or Chi squared test.

SD, standard deviation; (Q1–Q3), interquartile range; BMI, body mass index; SBP, systolic blood pressure; HbA1c, glycated hemoglobin, DBP, Diastolic blood pressure; HDL-c, high-density lipoprotein cholesterol; LDL-c, low-density lipoprotein cholesterol; LDL-p, low-density lipoprotein particle; IDP, intermediate-density lipoprotein particle.

### Lipid indices associations with ICAS

In main analyses, presented in Table 2, where lipid measures were treated as continuous variables, we found that the adjusted OR of prevalent ICAS was comparable for both LDL-p and LDL-c. Specifically, LDL-p exhibited an OR (95%CI) of 1.40 (1.15–1.70 while LDL-c demonstrated an OR (95% CI) of 1.38 (1.13–1.68).

**Table 2:** Adjusted odds ratio for the presence of ICAS per 1 SD higher value of the lipid indices (Japanese men, aged 40–79, SESSA).

	OR (95% CI)
LDL-c, mg/dL	1.38 (1.13–1.68)
LDL-p, nmol/L	1.40 (1.15–1.70)
Large LDL-p, nmol/L	1.10 (0.93–1.31)
Small LDL-p, nmol/L	1.26 (1.04–1.52)
IDL, nmol/L	1.06 (0.90–1.25)

Odds ratios were adjusted for age, body mass index, smoking status, drinking status, hypertension, diabetes mellitus, and dyslipidemia (base model).

OR, odds ratio; CI, confidence interval, LDL-c, low-density lipoprotein cholesterol; LDL-p, low-density lipoprotein particle; IDP, intermediate-density lipoprotein particle.

In sensitivity analyses, detailed in Table 3, the third (Q1) and four (Q4) quartiles of LDL-c and LDL-p were associated with prevalent ICAS. For LDL-c, ORs (95% CIs) for prevalent ICAS in the Q3 and Q4, in reference to Q1, were 2.87 (1.69–4.86) and 2.76 (1.61–4.71), respectively, after full-adjustment. Similarly, for LDL-p, OR (95%CI) of presence of ICAS in Q3 and Q4 were 2.60 (1.53–4.38) and 2.98 (1.76–5.03), respectively.

**Table 3:** Adjusted OR for the presence of ICAS across the Quartile of Lipid Indices (Japanese Men, aged 40–79, SESSA).

	Q1	Q2	Q3	Q4
	Range			
	Adjusted OR (95% CI)			
LDL-c, mg/dL	31–106.8	107.2–123.4	123.6–144.6	144.8–275.8
	Ref	1.68 (0.98–2.91)	2.87 (1.69–4.86)	2.76 (1.61–4.71)
LDL-p, nmol/L	297–1021	1023–1278	1280–1557	1558–3156
	Ref	1.55 (0.90–2.65)	2.60 (1.53–4.38)	2.98 (1.76–5.03)
Large LDL-p, nmol/L	0–422	423–617	618–815	817–1795
	Ref	1.09 (0.67–1.78)	0.94 (0.57–1.54)	1.25 (0.77–2.04)
Small LDL-p, nmol/L	0–108	109–539	541–856	858–1844
	ref	1.22 (0.72–2.04)	1.59 (0.94–2.69)	2.35 (1.40–3.92)
IDL, nmol/L	0–43	44–98	99–169	170–654
	ref	1.48 (0.91–2.39)	1.23 (0.75–2.03)	1.41 (0.86–2.32)

Odds ratios were adjusted for age, body mass index, smoking status, drinking status, hypertension, diabetes mellitus, and dyslipidemia (base model).

OR, odds ratio; CI, confidence interval, LDL-c, low-density lipoprotein cholesterol; LDL-p, low-density lipoprotein particle; IDP, intermediate-density lipoprotein particle.

The association between LDL-p and ICAS remained statistically significant after adjusting for LDL-c, as shown in Supplemental Table 1. After adjusting for LDL-p, the association between LDL-c and ICAS lost significance.

### Discordance of LDL-c with LDL-p and ICAS

The categorization of the study participants’ characteristics is presented in Table 4, based on the concordance–discordance analysis of LDL-c and LDL-p. It's important to note that participants lipid profiles fall into one of four groups, as depicted in Fig. 1. Figure 1 illustrated that the concordance group exhibits a higher prevalence in the upper right and lower left quadrants. The discordance group, which comprised 20.3% of the total population, included discordance “low LDL-c–high LDL-p” and discordance “high LDL-c–low LDL-p”. The group characterized by the discordance “low LDL-c–high LDL-p”, including approximately 10.7% of the study population, demonstrated the highest prevalence of ICAS, as shown in Table 4. The discordance “low LDL-c–high LDL-p” group demonstrated a larger tendency for increased alcohol consumption, elevated triglyceride levels, and lower HDL cholesterol levels. In addition, they had a higher prevalence of dyslipidemia, as well as a marginally higher prevalence of diabetes and hypertension in comparison to the other groups outlined in Table 4.

**Table 4:** Characteristic of study participants within the groups of concordance–discordance between LDL-c and LDL-p, based on their median cut-off point.

	Concordance–discordance group, n (%)				*p*-value
	Discordance “low LDL-c–high LDL-p”, 76 (10.7)	Discordance “high LDL-c–low LDL-p”, 68 (9.6)	Concordance “high LDL-c–high LDL-p”, 280 (39.4)	Concordance “low LDL-c–low LDL-p”, 287 (40.3)	
ICAS, n (%)	30 (39.5)	22 (32.4)	101 (36.0)	52 (18.1)	< 0.001
Age, years	62.6 (56.6–72.4)	66.6 (60.6–71.4)	63.8 (58.2–70.3)	64.1 (58.7–71.2)	0.457
Smoking status, n (%)					0.358
Current	30 (39.5)	19 (27.9)	83 (29.6)	77 (26.8)	
Past	32 (42.1)	32 (47.1)	147 (52.5)	151 (52.6)	
Never	14 (18.4)	17 (25)	50 (17.9)	59 (20.6)	
Drinking status, n (%)					0.001
Current	69 (90.8)	53 (77.9)	200 (71.4)	241 (84.0)	
Past	2 (2.6)	3 (4.4)	23 (8.2)	9 (3.1)	
Never	5 (6.6)	12 (17.7)	57 (20.4)	37 (12.9)	
BMI, kg/m^2^	24.5 ± 3	22.4 ± 2.5	24.3 ± 2.7	23 ± 2.9	0.425
SBP, mmHg	137 ± 17.3	134 ± 19.2	136.5 ± 17.8	134.6 ± 18.1	0.847
DBP, mmHg	81 ± 11.3	78.7 ± 10.8	80.5 ± 10.4	79.6 ± 11.1	0.714
Hypertension, n (%)	50 (65.8)	31 (45.6)	149 (53.2)	144 (50.2)	0.061
HbA1c, %	5.6 (5.3–6.1)	5.4 (5.1–5.7)	5.4 (5.2–5.9)	5.4 (5.1–5.8)	0.332
Glucose, mg/dL	104 (93–113)	94.5 (89.5–102)	98 (91–108)	97 (91–106)	0.425
Diabetes mellitus, n (%)	22 (29.0)	7 (10.3)	62 (22.1)	64 (22.3)	0.054
Dyslipidemia, n (%)	47 (61.8)	9 (13.2)	119 (42.5)	93 (32.4)	< 0.001
Total cholesterol, mg/dL	198.9 ± 25.1	225 ± 17.8	233 ± 27.1	188 ± 24.5	< 0.001
Triglycerides, mg/dL	125.5 (87.5–185.5)	86.5 (62.5–102)	121.5 (91–157)	90 (67–144)	0.005
HDL-c, mg/dL	50.97 ± 12.4	69.33 ± 16.5	53.5 ± 13.5	64.94 ± 18.24	0.001
LDL-c, mg/dL	115.3 ± 8.0	137 ± 10.5	153.8 ± 21.6	99.6 ± 17.9	< 0.001
LDL-p, nmol/L	1465.2 ± 173.4	1167.5 ± 87.1	1648.8 ± 271.1	945.3 ± 208.8	< 0.001
IDL-p, nmol/L	128.1 ± 109.0	101.7 ± 91.4	134.9 ± 112.5	112.7 ± 97.6	0.025
Large LDL-p, nmol/L	479.3 ± 278.2	835.6 ± 239.2	701.1 ± 305.5	547.3 ± 261.8	< 0.001
Small LDL-p, nmol/L	857.7 ± 318.3	230.1 ± 202.9	812.8 ± 383.8	285.2 ± 262.0	< 0.001

Values are presented as mean ± SD for continuous variables with approximately normally distribution or by median (Q1–Q3) with skewed distribution and n (%) for categorical variables. Differences in characteristics were evaluated by using the ANOVA test, Kruskal–Wallis test, or Chi squared test.

SD, standard deviation; (Q1–Q3), interquartile range; BMI, body mass index; SBP, systolic blood pressure; HbA1c, glycated hemoglobin, DBP, Diastolic blood pressure; HDL-c, high-density lipoprotein cholesterol; LDL-c, low-density lipoprotein cholesterol; LDL-p, low-density lipoprotein particle; IDP, intermediate-density lipoprotein particle.

In Table 5, the participants with the discordance "low LDL-c–high LDL-p" had a substantially higher OR (95% CI) of 2.78 (1.55–5.00) for the risk of intracranial artery stenosis in the reference of the concordance "low LDL-c–low LDL-p" group. The OR for participants with concordance "high LDL-c–high LDL-p" was 2.56 (1.69–3.85). The discordance "high LDL-c–low LDL-p" had a lowest OR of 2.40 (1.29–4.46).

**Table 5:** Adjusted odds ratio for the presence of ICAS of the concordance–discordance between LDL-c and LDL-p groups (Japanese men, aged 40–79, SESSA).

Concordance–discordance groups	OR (95% CI)
Discordance “low LDL-c–high LDL-p”	2.78 (1.55–5.00)
Discordance “high LDL-c–low LDL-p”	2.40 (1.29–4.46)
Concordance “high LDL-c–high LDL-p”	2.56 (1.69–3.85)
Concordance “low LDL-c–low LDL-p”	(reference)

Odds ratios were adjusted for age, body mass index, smoking status, drinking status, hypertension, diabetes mellitus, and dyslipidemia (base model).

OR, odds ratio; CI, confidence interval, LDL-c, low-density lipoprotein cholesterol; LDL-p, low-density lipoprotein particle; IDP, intermediate-density lipoprotein particle.

## Discussion

In this cross-sectional study of presumably healthy Japanese men, we discovered positive associations between LDL-c, LDL-p, small LDL-p and the presence of ICAS, independent of other conventional cardiovascular disease risk factors. Moreover, the discordance defined according median concentrations of LDL-c with LDL-p was reached approximately 20.3%. Furthermore, among these discordance groups, ICAS risk was underestimated about 10.7% in the participants who had low LDL-c and high LDL-p.

ICAS of major cerebral arteries is one of the common causes of ischemic stroke^18^, which, in turn, is still one of the leading causes of death and disability in Asian populations, including Japanese^19^. Previous studies have frequently reported the associations of lipid parameters such as TG, TC, LDL-c, non-HDL-c, HDL-c, and corresponding lipid ratios with ICAS^20–26^. In our previous reports in the same Japanese men population, many known risk factors including LDL-c have been reported to be related to the prevalent ICAS^27^.

To the best of our current understanding, this study represents the initial investigation into the association between NMR-measured LDL-p profiles with ICAS.

Of lipid indices assessed in this study, LDL-p and LDL-c seemed to have comparative predictive values associated with ICAS. However, we found that the effects of LDL-c association with ICAS was comparatively weaker and not entirely independent of LDL-p. The findings of this study indicate that, among the two measures of low-density lipoprotein, LDL-p demonstrates more strength and robustness as an indicator for the presence of ICAS. Histologically, low-density lipoprotein cholesterol (LDL-c) has been commonly recognized as a reliable indicator of LDL and has consequently been considered an important biomarker for atherogenic lipoproteins. As a result, it has become the primary focus for interventions aimed at reducing lipid levels. Numerous studies have provided evidence supporting the crucial role of LDL-c as a causal risk factor for vascular disorders, including coronary heart disease and ischemic stroke. Furthermore, the importance of LDL-c has been further emphasized by the observed reduction in major vascular events when treated with statins, which effectively lower LDL-c levels^28^. However, despite achieving the recommended LDL-c targets, the ICAS risk remains substantial in many patients^4^, which, in part, could be explained by other lipids. In order to enhance the predictability of lipid profiles, in addition to LDL-c, there has been considerable focus on other non-conventional lipid parameters. This study provides more evidence supporting the potential utility of LDL-p as a potential predictor for the treatment of intracranial atherosclerotic stenosis (ICAS), in addition to LDL-c.

LDL is comprised of a diverse array of particles that exhibit distinct physicochemical and metabolic properties. LDL-p contains a lipid core primarily composed of neutral lipids, including cholesterol esters and triglycerides, it is also recognized that free cholesterol is distributed throughout the particle found on the surface or shell of the lipoprotein. These particles also possess a shell composed of phospholipids and proteins, with the prominent presence of apolipoprotein B^29^. LDL-p serves as an alternative measure to LDL-c, exhibiting a strong correlation between both of them. However, it is important to note that the quantity of cholesterol transported by each LDL-p particle may differ. Elevated levels of circulating LDL-p have been associated with an increased likelihood of these particles causing harm to the artery wall and infiltrating the intimal layer. Once within the intimal layer, these particles undergo oxidation and trigger diverse processes involved in the formation of arterial plaques. LDL-p that possess small size have enhanced ability to infiltrate the subendothelial region of blood vessels, where macrophages subsequently absorb them. This process speeds up the development of foam cells. It is worth mentioning that the association between LDL-p size and the risk of developing atherosclerosis is influenced by the number of LDL-p. The association between LDL size and coronary heart disease loses its causative nature when the LDL-p quantity is within normal range^30^. The inquiry of the reliability of LDL-p as a marker for plaque formation, as well as the potential simultaneous elevation of LDL-p levels due to other atherosclerosis-related processes, is still in an early stage and necessitates additional research to obtain definitive conclusions.

Furthermore, a notable subgroup of individuals with optimally controlled LDL-c levels experience cardiovascular events^31^. As a result, there has been a suggestion that certain individuals may be at higher risk due to increased amounts of alternative lipid-measured, LDL-p, which may not be easily identifiable from their LDL-c values. Consequently, the risk in these individuals may be underestimated based solely on their LDL-c.

It is essential to engage in a discussion on some limitations. Initially, it is important to note that the study did not encompass women. However, focusing on the male population is significant due to the higher prevalence of cardiovascular diseases, such as stroke, among men in Asian countries like Japan. Additionally, we acknowledge the limitation of using a single measurement taken 6 years prior to the MRI scan. This time gap may introduce variability in lipid profiles due to lifestyle changes such as physical activity or dietary habits during the study period. Furthermore, we did not analyze clinical outcomes, such as incidence of cardiovascular events or mortality rates. This limits our ability to assess the direct impact of lipid profiles on actual health outcomes.

In conclusion, our study provides convincing evidence that, in addition to the conventional LDL-c, NMR-measured LDL-p and small LDL-p are associated with the development of ICAS in a population-based study of healthy Japanese men. However, if we rely solely on LDL-c, especially when LDL-c is within normal ranges, we will underestimate the residual prevalence of ICAS by 10.7% when LDL-c and LDL-p are discordant. Therefore, the results of this investigation support the use of alternative lipid measured among discordant male group.

### Supplementary Information

Supplementary Information.

## Data Availability

The dataset examined in this study is not available to the public due to the inclusion of individuals' personal information. The institutional review board at Shiga University of Medical Science will review every request, and researchers will be granted access to the data based on the approved conditions. Corresponding authors should be contacted if someone wants to request the data from this study.

## References

[1] Gorelick PB, Wong KS, Bae HJ, Pandey DK (2008). Large artery intracranial occlusive disease: A large worldwide burden but a relatively neglected frontier. Stroke.

[2] Koo J (2015). The latest information on intracranial atherosclerosis: Diagnosis and treatment. Interv. Neurol..

[3] Hero C, Svensson AM, Gidlund P, Gudbjörnsdottir S, Eliasson B, Eeg-Olofsson K (2016). LDL cholesterol is not a good marker of cardiovascular risk in Type 1 diabetes. Diabet. Med..

[4] Derdeyn CP, Chimowitz MI, Lynn MJ, Fiorella D, Turan TN, Janis LS (2014). Aggressive medical treatment with or without stenting in high-risk patients with intracranial artery stenosis (SAMMPRIS): The final results of a randomised trial. Lancet.

[5] Otvos JD, Mora S, Shalaurova I, Greenland P, Mackey RH, Goff DC (2011). Clinical implications of discordance between low-density lipoprotein cholesterol and particle number. J. Clin. Lipidol..

[6] Zaid M, Fujiyoshi A, Miura K, Abbott RD, Okamura T, Takashima N (2015). High-density lipoprotein particle concentration and subclinical atherosclerosis of the carotid arteries in Japanese men. Atherosclerosis.

[7] Zaid M, Miura K, Fujiyoshi A, Abbott RD, Hisamatsu T, Kadota A (2016). Associations of serum LDL particle concentration with carotid intima-media thickness and coronary artery calcification. J. Clin. Lipidol..

[8] Vu T, Fujiyoshi A, Hisamatsu T, Kadota A, Zaid M, Segawa H (2021). Lipoprotein particle profiles compared with standard lipids in the association with subclinical aortic valve calcification in apparently healthy Japanese men. Circ. J..

[9] Kadota A, Miura K, Okamura T, Fujiyoshi A, Ohkubo T, Kadowaki T (2013). Carotid intima-media thickness and plaque in apparently healthy Japanese individuals with an estimated 10-year absolute risk of CAD death according to the Japan Atherosclerosis Society (JAS) guidelines 2012: The Shiga Epidemiological Study of Subclinical Atherosclerosis (SESSA). J. Atheroscler. Thromb..

[10] Ueshima H, Kadowaki T, Hisamatsu T, Fujiyoshi A, Miura K, Ohkubo T (2016). Lipoprotein-associated phospholipase A2 is related to risk of subclinical atherosclerosis but is not supported by Mendelian randomization analysis in a general Japanese population. Atherosclerosis.

[11] Cuschieri S (2019). The STROBE guidelines. Saudi J. Anaesth..

[12] Friedewald WT, Levy RI, Fredrickson DS (1972). Estimation of the concentration of low-density lipoprotein cholesterol in plasma, without use of the preparative ultracentrifuge. Clin. Chem..

[13] Jeyarajah EJ, Cromwell WC, Otvos JD (2006). Lipoprotein particle analysis by nuclear magnetic resonance spectroscopy. Clin. Lab. Med..

[14] Otvos JD, Jeyarajah EJ, Cromwell WC (2002). Measurement issues related to lipoprotein heterogeneity. Am. J. Cardiol..

[15] Kashiwagi A, Kasuga M, Araki E, Oka Y, Hanafusa T, Ito H (2012). International clinical harmonization of glycated hemoglobin in Japan: From Japan Diabetes Society to National Glycohemoglobin Standardization Program values. J. Diabetes Investig..

[16] Samuels OB, Joseph GJ, Lynn MJ, Smith HA, Chimowitz MI (2000). A standardized method for measuring intracranial arterial stenosis. AJNR Am. J. Neuroradiol..

[17] Mora S, Buring JE, Ridker PM (2014). Discordance of low-density lipoprotein (LDL) cholesterol with alternative LDL-related measures and future coronary events. Circulation.

[18] Famakin BM, Chimowitz MI, Lynn MJ, Stern BJ, George MG (2009). Causes and severity of ischemic stroke in patients with symptomatic intracranial arterial stenosis. Stroke.

[19] Takashima N, Arima H, Kita Y, Fujii T, Tanaka-Mizuno S, Shitara S (2020). Long-term survival after stroke in 1.4 million Japanese population: Shiga stroke and heart attack registry. J. Stroke.

[20] Ma YH, Leng XY, Dong Y, Xu W, Cao XP, Ji X (2019). Risk factors for intracranial atherosclerosis: A systematic review and meta-analysis. Atherosclerosis.

[21] Kim BS, Jung HS, Bang OY, Chung CS, Lee KH, Kim GM (2010). Elevated serum lipoprotein(a) as a potential predictor for combined intracranial and extracranial artery stenosis in patients with ischemic stroke. Atherosclerosis.

[22] Park JH, Hong KS, Lee EJ, Lee J, Kim DE (2011). High levels of apolipoprotein B/AI ratio are associated with intracranial atherosclerotic stenosis. Stroke.

[23] Wang S, Wang X, Zhao Y, Ji X, Sang S, Shao S (2020). Characterizing lipid profiles associated with asymptomatic intracranial arterial stenosis in rural-dwelling adults: A population-based study. J. Clin. Lipidol..

[24] Li X, Wang A, Wang J, Wu J, Wang D, Gao X (2017). Association between high-density-lipoprotein-cholesterol levels and the prevalence of asymptomatic intracranial arterial stenosis. Sci. Rep..

[25] Guo J, Wang A, Wang Y, Liu X, Zhang X, Wu S (2021). Non-traditional lipid parameters as potential predictors of asymptomatic intracranial arterial stenosis. Front. Neurol..

[26] Suri MF, Qiao Y, Ma X, Guallar E, Zhou J, Zhang Y (2016). Prevalence of intracranial atherosclerotic stenosis using high-resolution magnetic resonance angiography in the general population: The Atherosclerosis Risk in Communities Study. Stroke.

[27] Shitara S, Fujiyoshi A, Hisamatsu T, Torii S, Suzuki S, Ito T (2019). Intracranial artery stenosis and its association with conventional risk factors in a general population of Japanese men. Stroke.

[28] Cholesterol Treatment Trialists C (2012). The effects of lowering LDL cholesterol with statin therapy in people at low risk of vascular disease: Meta-analysis of individual data from 27 randomised trials. Lancet.

[29] Wolska A, Remaley AT (2020). Measuring LDL-cholesterol: What is the best way to do it?. Curr. Opin. Cardiol..

[30] Allaire J, Vors C, Couture P, Lamarche B (2017). LDL particle number and size and cardiovascular risk: Anything new under the sun?. Curr. Opin. Lipidol..

[31] Sampson UK, Fazio S, Linton MF (2012). Residual cardiovascular risk despite optimal LDL cholesterol reduction with statins: The evidence, etiology, and therapeutic challenges. Curr. Atheroscler. Rep..

