# Supplementary material for: Low-density lipoprotein particle profiles compared with standard lipids measurements in the association with asymptomatic intracranial artery stenosis
Source: Sci Rep. 2024 May 10;14:10765. doi: 10.1038/s41598-024-59523-4 (PMC11087462; doi:10.1038/s41598-024-59523-4)
Supplement: Supplementary file 1 — Supplementary Information. [file 41598_2024_59523_MOESM1_ESM.pdf]

Supplementary

Title: Low-density Lipoprotein Particle Profiles compared with Standard Lipids Measurements in the association with Asymptomatic Intracranial Artery Stenosis

Authors, Affiliations, Contact Information

Thien Vu [1, 2], Yuichiro Yano [1], Tai Pham [1, 3, 4], Rajib Mondal [1], Mizuki Ohashi [1], Kaori Kitaoka [1], Mohammad Moniruzzaman [1], Sayuki Torii [1, 5], Akihiko Shiino [6], Atsushi Tsuji [7], Takashi Hisamatsu [5, 8], Tomonori Okamura [9], Keiko Kondo [1, 5], Aya Kadota [1], Yoshiyuki Watanabe [10], Kazuhiko Nozaki [11], Hirotsugu Ueshima [1], Katsuyuki Miura [1, 5, *].

[1] NCD Epidemiology Research Center, Shiga University of Medical Science, Otsu, Shiga, Japan. [2] Department of Cardiac Surgery, Cardiovascular Center, Cho Ray hospital, Ho Chi Minh City, Vietnam. [3] Department of Oriental Internal Medicine, Faculty of Traditional Medicine, University of Medicine and Pharmacy, Ho Chi Minh City, Viet Nam. [4] Department of Geriatrics, Gia-Dinh People's Hospital, Ho Chi Minh City, Viet Nam. [5] Department of Public Health, Shiga University of Medical Science, Otsu, Shiga, Japan. [6] Molecular Neuroscience Research Center, Shiga University of Medical Science, Otsu, Shiga, Japan. [7] Department of Neurosurgery, Shiga University of Medical Science, Otsu, Shiga, Japan. [8] Department of Public Health Okayama University Graduate School of Medicine, Dentistry and Pharmaceutical Sciences Okayama, Japan. [9] Department of Hygiene and Public Health, Keio University School of Medicine, Tokyo, Japan. [10] Department of Radiology, Shiga University of Medical Science, Otsu, Shiga, Japan. [11] Higashi-Ohmi General Medical Center, Higashiomi City, Shiga, Japan.

Corresponding authors:

Katsuyuki Miura

- NCD Epidemiology Research Center, Shiga University of Medical Science, Otsu, Shiga, Japan.
- Department of Public Health, Shiga University of Medical Science, Otsu, Shiga, Japan.

  miura@belle.shiga-med.ac.jp

Number of supplementary materials:

- **SESSA Research Group Members**
- **Supplementary Table 1**: Odds ratio for the presence of intracranial artery stenosis per 1-SD higher value of lipid indices
- **STROBE statement**.

SESSA Research Group Members

Co-chairpersons: Hirotsugu Ueshima (Department of Public Health, Shiga University of Medical Science, Otsu, Shiga), Katsuyuki Miura (Department of Public Health, Shiga University of Medical Science, Otsu, Shiga).

Research members: Akira Fujiyoshi (Wakayama Medical University); Minoru Horie, Takashi Yamamoto, Hideki Hayashi, Yasutaka Nakano, Emiko Ogawa, Hiroshi Maegawa, Itsuko Miyazawa, Kiyoshi Murata, Kazuhiko Nozaki, Ikuo Toyama, Akihiko Shiino, Akira Andoh, Teruhiko Tsuru, Hisakazu Ogita, Akio Shimizu, Naoko Miyagawa, Atsunori Kashiwagi, Aya Kadota, Naoyuki Takashima (Kindai University Faculty of Medicine); Takashi Kadowaki, Sayaka Kadowaki, Sayuki Torii, Robert D. Abbott, Keiko Kondo, Maryam Zaid, Sentaro Suzuki, Takahiro Ito, Ayako Kumimura (Shiga University of Medical Science, Otsu, Shiga); Yoshihiko Nishio (Kagoshima University, Kagoshima); Kenichi Mitsunami (Vories Memorial Hospital, Omihachiman, Shiga); Toru Kita, Takeshi Kimura, Yasuharu Tabara (Kyoto University, Kyoto); Yasuyuki Nakamura (Ryukoku University, Kyoto); Tomonori Okamura (Keio University, Tokyo); Akira Sekikawa, Emma JM Barinas-Mitchell (University of Pittsburgh, Pittsburgh, PA, USA); Takayoshi Ohkubo (Teikyo University, Tokyo); Yoshikuni Kita (Tsuruga Nursing University, Tsuruga, Fukui); Yoshitaka Murakami (Toho University, Ota, Tokyo); Atsushi Hozawa (Tohoku University, Sendai, Miyagi); Nagako Okuda (University of Human Arts and Sciences, Iwasuki-ku, Saitama); Aya Higashiyama, Seiko Ohno (Research and Development Initiative Center, National Cerebral and Cardiovascular Center, Suita, Osaka); Shinya Nagasawa (Kanazawa Medical University, Kanazawa, Ishikawa); Takashi Hisamatsu (Okayama University, Okayama); Masahiro Yamazoe (Tokyo Medical and Dental University, Tokyo); Yoshino Saito (Aino University, Takatsuki, Osaka); Daniel Edmundowicz (Temple University); Hisatomi Arima, Atsushi Satoh (Fukuoka University, Fukuoka); Koichiro Azuma (Keio University School of Medicine, Tokyo); Masahiko Yanagita (Doshisha University, Kyoto); Tatsuya Sawamura (Shinshu University, Nagano); Michiya Igase (Ehime University, Matsuyama, Ehime).

Supplementary Table 1: Odds ratio for the presence of intracranial artery stenosis per 1SD higher value of lipid indices

Lipid indices	Model	OR (95% CI)
LDL-c, mg/dL	base model + LDL-p	1.07 (0.78-1.46)
LDL-p, nmol/L	base model + LDL-c	1.40 (1.01-1.93)

Odds ratios were adjusted for age, body mass index, smoking status, drinking status, hypertension, diabetes mellitus, and dyslipidemia (base model).

Abbreviations: OR, odds ratio; CI, confidence interval; LDL-c, low-density lipoprotein cholesterol; LDL-p, low-density lipoprotein particle; IDP, intermediate-density lipoprotein particle.

STROBE Statement - Checklist of items that should be included in reports of ***cross-sectional studies***

	Item No	Recommendation	Page No
**Title and abstract**	1	(*a*) Indicate the study's design with a commonly used term in the title or the abstract	p.II
		(*b*) Provide in the abstract an informative and balanced summary of what was done and what was found	p.II
**Introduction**			
Background/rationale	2	Explain the scientific background and rationale for the investigation being reported	p.III
Objectives	3	State specific objectives, including any prespecified hypotheses	p.III
**Methods**			
Study design	4	Present key elements of study design early in the paper	p.IV
Setting	5	Describe the setting, locations, and relevant dates, including periods of recruitment, exposure, follow-up, and data collection	p.IV
Participants	6	(*a*) Give the eligibility criteria, and the sources and methods of selection of participants	p.IV
Variables	7	Clearly define all outcomes, exposures, predictors, potential confounders, and effect modifiers. Give diagnostic criteria, if applicable	p.IV, V, VI
Data sources/ measurement	8*	For each variable of interest, give sources of data and details of methods of assessment (measurement). Describe comparability of assessment methods if there is more than one group	p.IV, V, VI
Bias	9	Describe any efforts to address potential sources of bias	p.V
Study size	10	Explain how the study size was arrived at	p.IV
Quantitative variables	11	Explain how quantitative variables were handled in the analyses. If applicable, describe which groupings were chosen and why	p.IV, V, VI
Statistical methods	12	(*a*) Describe all statistical methods, including those used to control for confounding	p.VII, VIII
		(*b*) Describe any methods used to examine subgroups and interactions	p.IV
		(*c*) Explain how missing data were addressed	p.IV
		(*d*) If applicable, describe analytical methods taking account of sampling strategy	
		(*e*) Describe any sensitivity analyses	p.VIII
**Results**			

Participants	13*	(a) Report numbers of individuals at each stage of study—eg numbers potentially eligible, examined for eligibility, confirmed eligible, included in the study, completing follow-up, and analysed	p.VIII
		(b) Give reasons for non-participation at each stage	
		(c) Consider use of a flow diagram	
Descriptive data	14*	(a) Give characteristics of study participants (eg demographic, clinical, social) and information on exposures and potential confounders	p.VIII
		(b) Indicate number of participants with missing data for each variable of interest	
Outcome data	15*	Report numbers of outcome events or summary measures	p.VIII
Main results	16	(*a*) Give unadjusted estimates and, if applicable, confounder-adjusted estimates and their precision (eg, 95% confidence interval). Make clear which confounders were adjusted for and why they were included	p.VIII
		(*b*) Report category boundaries when continuous variables were categorized	
		(*c*) If relevant, consider translating estimates of relative risk into absolute risk for a meaningful time period	
Other analyses	17	Report other analyses done—eg analyses of subgroups and interactions, and sensitivity analyses	p. IX
**Discussion**			
Key results	18	Summarise key results with reference to study objectives	p.X
Limitations	19	Discuss limitations of the study, taking into account sources of potential bias or imprecision. Discuss both direction and magnitude of any potential bias	p.XII, XIII
Interpretation	20	Give a cautious overall interpretation of results considering objectives, limitations, multiplicity of analyses, results from similar studies, and other relevant evidence	p.X, XI, XII, XIII
Generalisability	21	Discuss the generalisability (external validity) of the study results	p.XII
**Other information**			
Funding	22	Give the source of funding and the role of the funders for the present study and, if applicable, for the original study on which the present article is based	p.XIV

*Give information separately for exposed and unexposed groups.

Note: An Explanation and Elaboration article discusses each checklist item and gives methodological background and published examples of transparent reporting. The STROBE checklist is best used in conjunction with this article (freely available on the Web sites of PLoS Medicine at http://www.plosmedicine.org/, Annals of Internal Medicine at http://www.annals.org/, and Epidemiology at http://www.epidem.com/). Information on the STROBE Initiative is available at www.strobe-statement.org.
